# Virtual versus in-person ROSE program (La Luz) as universal prevention for perinatal depression: Protocol for a randomized controlled trial in a safety net hospital

**DOI:** 10.1016/j.cct.2025.107988

**Published:** 2025-06-15

**Authors:** Daphne Y. Liu, Nicholas S. Perry, Catherine H. Demers, Jennifer S. Hyer, Anely Alamo, Paige Vuksanovich, Nandi Dube, Erin R. Flanagan, Robert J. Gallop, Galena K. Rhoades, Elysia Poggi Davis

**Affiliations:** a University of Denver, 2155 S Race St, Denver, CO 80210, USA; b University of Colorado School of Medicine, Anschutz Medical Campus, 1890 N Revere Ct, Aurora, CO 80045, USA; c Denver Health, 777 Bannock St., Denver, CO 80204, USA; d West Chester University, 700 South High St, West Chester, PA 19383, USA; e University of California, Irvine, Department of Pediatrics, 1001 Health Sciences Road, Irvine, CA 92697, USA

**Keywords:** Perinatal depression, ROSE, Interpersonal psychotherapy, Hispanic/Latina/x, Randomized controlled trial, Implementation science

## Abstract

Perinatal depression disproportionally affects underserved communities, who need effective and accessible prevention programs. Reach Out, Stay strong, Essentials for new mothers (ROSE) has demonstrated effectiveness in preventing postpartum depression in underserved populations when delivered in person. This ongoing randomized controlled trial (RCT) tests the novel virtual implementation of ROSE as universal prevention in a Federally Qualified Health Center setting. We adopted the name, La Luz, for ROSE to be culturally relevant to our population. Pregnant individuals <30 gestational weeks (target *N* = 900) are randomized to either the virtual or in-person La Luz program, which consists of four 90-min group sessions. Groups are offered in English and Spanish, based on participants’ preference. Participants complete surveys at seven timepoints: before program, 28 gestational weeks, 35 gestational weeks, and 6-weeks, 3-months, 6-months, and 12-months postpartum. Primary outcomes are postpartum depression severity measured by the 20-item Symptom Checklist (SCL-20) and 10-item Center of Epidemiologic Studies Depression Scale (CESD-10). We will perform intent-to-treat analysis to test whether virtual La Luz is non-inferior to in-person La Luz. This study also evaluates implementation process and outcomes of both modalities of La Luz to inform the development of an implementation toolkit. This RCT contributes to our knowledge of perinatal depression prevention and addresses access barriers for underserved populations. If effectiveness of virtual La Luz is demonstrated, the new evidence for a scalable low-cost prevention can be used to reduce perinatal depression and offer accessible treatment options for people who face significant barriers to care. Clinicaltrials.gov registration identifier: NCT05766475.

## 1. Introduction

Perinatal depression, the experience of elevated depressive symptoms or the occurrence of a major depressive disorder during the perinatal period (i.e., pregnancy to one year postpartum), is one of the most common complications of pregnancy [1]. It is estimated that 27.4 % of pregnant individuals globally experience elevated depressive symptoms, and 17.0 % meet diagnostic criteria for a major depressive episode, during the perinatal period [2]. Suicidal thoughts and behaviors can occur as a symptom of perinatal depression and are a leading cause of maternal morbidity and mortality [3,4]. Perinatal depression has far-reaching implications for the next generation, including premature birth, developmental delays, and vulnerability to psychopathology [5–10]. Rates of depression vary based on sociocultural factors. For example, depression is more prevalent among pregnant individuals exposed to contextual risk factors such as poverty and racism [11,12]. Systemic stressors, including systemic racism, trauma, and economic inequality, disproportionately affect those who identify as Black or African American, Hispanic/Latine, American Indian, Alaska Native, or multiple races or ethnicities, contributing to elevated rates of perinatal depression [13–15]. Further, epidemiological data indicate that Hispanic/Latine women report more persistent and severe depression [16,17]. As the population of individuals who identify as racial and ethnic minorities is growing in the United States, the health disparities experienced by minoritized pregnant individuals and their families represent an increasingly urgent public health concern.

Considering the risk for suicide and long-term adverse impact of perinatal depression, prevention efforts are critically needed [18]. Reducing perinatal depression can prevent the numerous negative outcomes it can cause for both the pregnant individual and the next generation [8,19,20]. The intergenerational benefits of addressing perinatal depression support the need for inexpensive, widely available perinatal depression prevention programs. Although several perinatal depression interventions have shown efficacy in reducing depression during pregnancy [21,22] and postpartum [23–25], it is critical to increase their reach to communities in need, particularly those at elevated risk for perinatal depression and those who experience barriers to care [11,12]. Thus, an essential next step is to ensure that evidence-based prevention programs are readily available and accessible to minoritized communities to reduce perinatal depression [26].

The Reach Out, Stay strong, Essentials for new mothers (ROSE) program [23] is a well-established interpersonal therapy-oriented group intervention for the prevention of postpartum depression [23,24]. ROSE consists of four 90-min, weekly group sessions and one individual postpartum booster session. Content addresses social support, role transition to motherhood, communication skills, and psychoeducation on postpartum depression [23,27]. Among those identified as being at risk for postpartum depression, ROSE is effective at preventing the onset of postpartum depression [23,24,27,28], with an average reduction in risk of 29 % [29], and effectiveness has been sustained through 12-month follow-up [23]. This clear and abundant evidence led the United States Preventive Services Task Force to name ROSE as one of only two established interventions effective at reducing postpartum depression [29]. Critically, ROSE has demonstrated the benefits of ROSE in preventing postpartum depression in populations that are under-served in mental health treatment, including those living in poverty as well as Black or African-American and Hispanic/Latine individuals [23,24,27,28]. Thus, there is strong evidence supporting ROSE as a postpartum depression prevention program, including among those who face health disparities. Notably, because prior trials have only tested ROSE among people who are identified as at risk for postpartum depression based on prenatal screening [23,24,27,28], implementation of ROSE as universal prevention (i.e., delivered to all pregnant people regardless of risk) remains to be tested.

Although effective perinatal depression preventions such as ROSE exist and have demonstrated promising evidence among racially, ethnically and socioeconomically marginalized populations [29], disparities in psychosocial treatment (e.g., limited access, substandard quality of care) reduce the access to effective interventions for minoritized populations [30–32]. This problem is especially apparent among Black and Hispanic/Latine individuals, who face a persistent and increasingly widening disparity in access to mental health care compared to White individuals [33,34] despite their high depression rates depression [15,35]. Although socioeconomic factors (e.g., income, insurance coverage) contribute to mental health care access, disparities remain to exist among racial and ethnic minoritized groups after accounting for these socioeconomic factors [36–38]. Additional barriers to treatment include stigma associated with mental illness, lack of culturally responsive and linguistically appropriate interventions or providers, challenges navigating the healthcare system, logistical barriers (e.g., lack of childcare), and experiences of racism and other forms of discrimination [30,36]. These barriers are particularly evident among Hispanic/Latine and Black populations [30] and contribute to a wide treatment gap among people most at-risk for perinatal depression [32,39].

The preventive intervention (i.e., ROSE) tested in this study addresses many of these barriers by adopting a universal prevention approach, delivering culturally sensitive treatment in English and Spanish, and testing telehealth delivery. Offering ROSE as a universal prevention within the obstetric setting can help improve access by normalizing and de-stigmatizing participation in prevention programs, especially for those who experience elevated social stigma and fear of negative consequences associated with being identified as needing interventions (e.g., racially and ethnically minoritized individuals) [40]. Additionally, providing services in patients’ preferred language can help reduce barriers to care and attrition by easing communication, acknowledging the patient’s language and cultural background, and facilitating bonding and sense of community among people with shared language backgrounds [30,41]. In particular, the Hispanic and Latine population is the largest, and one of the fastest-growing, racial and ethnic minoritized groups in the United States [42], and over 43 million people speak Spanish at home [43]. Additionally, the Hispanic and Latine population has one of the highest fertility rates across various racial and ethnic groups in the United States [44]. As such, providing perinatal prevention programs in Spanish when preferred can promote acceptability and adoption of these programs among Spanish-speaking pregnant individuals. However, although ROSE is available in Spanish [45], the effectiveness of ROSE in Spanish has not been empirically tested.

Expanding ROSE through virtual delivery offers another transformative strategy to improve access of care in underserved populations [46,47]. To date, trials establishing the efficacy of ROSE have only been conducted in person [23,24,27,28,48,49]. Given its widespread scalability, telehealth has the potential to enhance mental health service delivery by reducing access barriers, particularly among minoritized groups [46,47]. For example, prior research with ROSE found that Black individuals identified transportation as a major barrier [49]. Specific to perinatal depression treatment, telehealth can be an effective means of intervention [50,51]. Thus, a preventive intervention delivered via telehealth is a potentially scalable, sustainable, and effective delivery model in real-world settings.

## 2. The current research

To optimize access to efficacious preventive interventions for perinatal depression, we are conducting a randomized controlled trial (RCT) of ROSE to test the comparative effectiveness of virtual versus in-person delivery. The study design offers ROSE as a brief, low-cost treatment as universal prevention within the obstetric setting, delivers it in one’s preferred language (English or Spanish), and compares telehealth and in-person services. We adopted a name that a local non-profit, Thriving Families, uses for their ROSE program, La Luz, as this name is more culturally relevant to the population we intend to enroll. This name was chosen because “Dar a Luz” is the Spanish phrase for “to give birth”, which also has a symbolic meaning of “bring light to life.” This RCT is being implemented at Denver Health, a community safety-net hospital and Federally Qualified Health Center that serves the largest proportion of uninsured and Medicaid patients in the Denver area [52]. Patients are English- and Spanish-speaking, predominantly Hispanic/Latine and socioeconomically disadvantaged. Denver Health delivers over 4300 babies each year, which accounts for approximately a third of all babies delivered in Denver [53]. Pregnant individuals are randomized to receive La Luz either (a) in person, delivered at Denver Health, or (b) virtually, delivered by the same staff via video conferencing.

This research has two main aims. The first aim is to test the effectiveness of virtual delivery of La Luz in reducing perinatal depression compared to in-person delivery. We hypothesize that La Luz administered virtually during pregnancy will show non-inferior benefits to reducing depression as in-person delivery. The second aim is to provide recommendations for sustainability and scalability to other perinatal healthcare settings. Implementation processes and outcomes (i.e., feasibility, acceptability, appropriateness, adoption, and barriers) will be examined qualitatively and quantitatively for both the in-person and virtual interventions. Additionally, the impact of the program on patients’ healthcare service utilization will be explored and the cost of the intervention will be documented. These data will be used to develop an implementation toolkit to support sustainability, scalability, and transportability to other healthcare settings.

## 3. Method

### 3.1. Participant enrollment

#### 3.1.1. Study population and recruitment site

We aim to recruit 900 pregnant individuals from Denver Health obstetrics clinics. Pregnant individuals (18 years or older) who are English or Spanish speaking and at less than 30 gestational weeks are eligible. Individuals at 30 or more gestational weeks are excluded due to insufficient time to complete ROSE prior to delivery. The target sample size was determined based on power analyses accounting for an estimated 20 % attrition rate (10 % during pregnancy and an additional 10 % postpartum) and our primary analyses testing inferiority of virtual to in-person La Luz (see below).

Denver Health is a Federally Qualified Health Center serving low resourced and underserved pregnant individuals. The obstetrics clinics at Denver Health serve individuals from the surrounding urban metropolitan Denver area. Denver is a racially and ethnically diverse city, which is reflected in the Denver Health patient population. Among all patients who received care from Denver Health in 2023, 49 % identify as Hispanic/Latine, 13 % Black or African American, 30 % non-Hispanic White, and 4 % Asian or Pacific Islander [54]. Spanish-speaking patients make up a substantial portion of the Denver Health patient population, and some patients speak only Spanish [55].

#### 3.1.2. Recruitment procedures

**Fig. 1.**
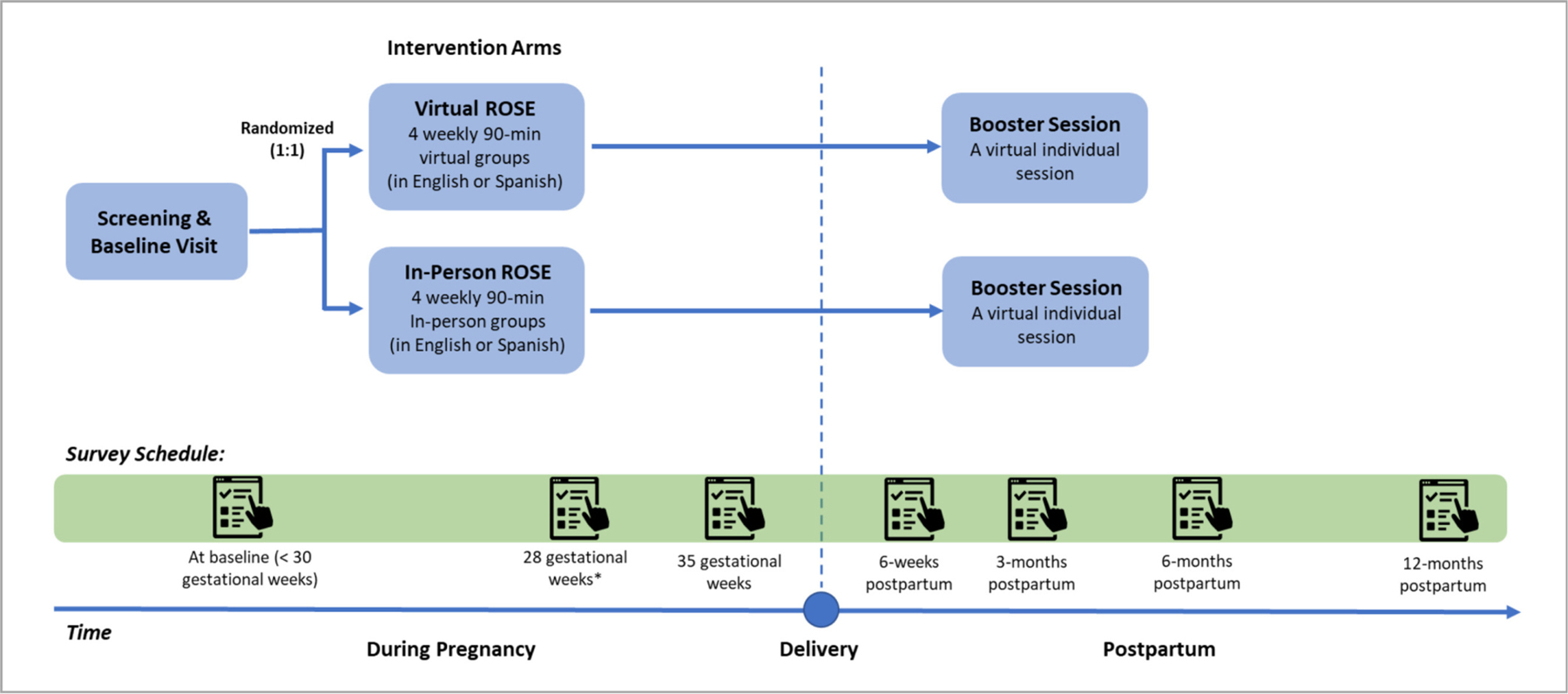
Overview of study flow. Note. This figure provides an overview of study procedures, including randomization, intervention, and timing of assessments. ROSE = Reach Out, Stay strong, Essentials for new mothers program. * If a participant is enrolled at 28 or 29 gestational weeks, they will receive their 28-gestational week survey 1–2 days after their baseline survey.

Recruitment began in March 2023 and is currently ongoing. Fig. 1 provides an overview of study procedures. On-site research staff screen prenatal visits scheduled at Denver Health for inclusion criteria (<30 gestational weeks, English or Spanish speaking) using electronic health records. Patients who meet inclusion criteria are approached by the bilingual (English/Spanish) on-site project staff at their prenatal visits or via phone calls. If interested, they are scheduled for their baseline study visit. All recruitment and study procedures were approved by the Institutional Review Board at the University of Denver. The current clinical trial was registered prior to the onset of participant enrollment (clinicaltrials.gov registration identifier: NCT05766475).

### 3.2. Baseline study visit and randomization

At their baseline study visit, participants complete informed consent with research staff and the baseline survey via REDCap over the phone or via Zoom videoconferencing. At the end of their baseline visit, participants are randomly assigned to one of the two conditions (virtual or in-person) using a secure and locked computer program and are scheduled for their first group session. Research staff are blind to participants’ condition assignment during survey administration and data analysis.

### 3.3. Study interventions

#### 3.3.1. Description of ROSE (Called La Luz in this Study)

ROSE is designed as four sessions delivered prenatally with a postpartum booster session [23]. The first session covers creating realistic expectations around the postpartum period and psychoeducation on PPD, the second session addresses strategies for managing the role transition to motherhood with a focus on identifying sources of support and increasing pleasurable activities, the third session provides education on relationship dynamics and how to communicate assertively with others, and the fourth is on understanding the barriers to assertiveness and planning for the future. Throughout all four sessions, there is an emphasis on learning relaxation practices and identifying pleasant activities as coping strategies for managing changes after childbirth.

#### 3.3.2. Virtual vs. in-person delivery

We planned for the first four sessions of La Luz to be delivered prenatally in groups of 6 to 20, with a projected average of 12 per group. The group program is 90 min weekly for four weeks. The same facilitators co-facilitate the in-person and virtual modalities and across languages. Participants in both conditions are provided with workbooks by mail after their initial study intake appointment. Participants can complete any missed session by attending the session of a future cohort in the same modality or schedule an individual make-up session with the facilitator by phone or video. Following the birth of the baby, the individual booster session (typically 30–45 min) is scheduled with one of the facilitators. The booster session is delivered virtually for all participants (in both groups).

For in-person groups, transportation is provided to reduce barriers to attendance. For virtual groups, tablets with Zoom are provided to participants who need a video conferencing device. Transportation and tablets are provided to ensure that neither transportation nor technology were an inequitable barrier that negatively impacted participants’ perception or participation in the in-person or virtual groups. Participants in both conditions receive $10 for every group session they attend via a reloadable Visa card.

#### 3.3.3. Provider training and fidelity

ROSE is designed to be a flexible, adaptable program that requires minimal training to deliver [56]. The five La Luz co-facilitators to date are bilingual psychologists or social workers experienced with working with the perinatal population. The facilitators are supervised by GKR, a licensed clinical psychologist and an experienced supervisor trained in ROSE, via monthly supervision meetings to ensure the quality and fidelity of intervention delivery. La Luz facilitators were trained in one of three ways (each took approximately four hours): (1) virtual training conducted by the developer of ROSE (two facilitators); (2) in-person training conducted by an experienced facilitator of La Luz at Thriving Families, a local non-profit that also offers La Luz, following the standard ROSE training protocol (two facilitators); or (3) online training available through the official ROSE program website [56] (one facilitator).

### 3.4. Evaluation of implementation process and outcomes

Evaluation of the implementation of La Luz will occur quantitatively and qualitatively in this study, guided by Proctor’s implementation outcomes framework [57] and the Integrated Promoting Action on Research Implementation in Health Services (i-PARIHS) model [58]. Proctor’s framework operationalizes the goals of quality evidence-based mental healthcare, including perceived feasibility, acceptability, and appropriateness of treatment, and costs of the intervention [57]. The i-PARIHS guides qualitative measurement of the implementation process and focuses on three elements that drive successful implementation of an intervention: innovation, recipients, and context [58]. Table 1 provides definitions of constructs of these two frameworks.

Approximately 30 participants (12–15 per delivery modality) will be recruited to complete focus groups (in English and Spanish) within 6 months of completing the fourth La Luz group session. Focus groups use a semi-structured in-depth format with open-ended questions and last approximately 60 min. The focus groups explore themes relevant to i-PARIHS constructs: 1) perceived challenges and barriers, including perceived cultural relevance and barriers, to session attendance and engagement with the intervention material and content; 2) perceived risks and benefits of the treatment modality received (i.e., in-person vs virtual) for perinatal depression; 3) perceived appropriateness and fit of the intervention and delivery modality to participants’ specific needs; and 4) perceived skills and benefits derived from the intervention. Focus groups will also be conducted with approximately 10 hospital providers, staff, and administrators after enrollment into the RCT ends; these focus groups assess constructs similar to those assessed in the participant focus groups regarding implementation drivers, with a focus on sustainability of the program in the setting after the research study ends.

### 3.5. Measures

#### 3.5.1. Measures overview and schedule

Participants complete self-report surveys via REDCap at seven assessment timepoints: before the program begins (baseline), at 28 gestational weeks, 35 gestational weeks, and at 6-weeks, 3-months, 6-months, and 12-months postpartum. Sociodemographic constructs are assessed at baseline and postpartum. Measures of mental health (e.g., depression severity) and other psychosocial constructs (e.g., social support) are assessed at each timepoint. Data are also collected from electronic health records for relevant constructs, including depressive symptoms and pregnancy-related information (e.g., gestational age, obstetric complications), and other health information (e.g., care utilization, medications). Participants receive $50 via a reloadable Visa card for each completed survey. They additionally receive a $50 bonus if they complete all three prenatal surveys and the 6-weeks postpartum survey, and another $50 bonus if they complete the 3-months, 6-months, and 12-months postpartum surveys.

#### 3.5.2. Primary and secondary outcome measures

##### Primary outcomes.

Depression symptoms are measured using (1) the 20-item Symptom Checklist (SCL-20) developed from the full Symptom Checklist-90-R [59], and (2) the 10-item Center of Epidemiologic Studies Depression Scale (CESD-10) shortened based on the original 20-item scale [60,61]. Both SCL-20 and CESD-10 are reliable and valid measures of depressive symptom severity, including with pregnancy participants [7,21,22,62]. For both scales, participants provided ratings using a 4-point scale on their symptoms for the past week (CESD-10) or month (SCL-20). Item scores for each scale are summed to generate a total score, with higher scores indicating higher depression severity. The SCL-20 and CESD-10 scores will be analyzed dimensionally as the primary outcomes of this RCT.

##### Secondary outcome.

Secondary analyses will involve evaluating depression assessed via the Edinburgh Postnatal Depression Scale (EPDS), which is widely used for screening maternal depression across the perinatal period [63]. Participants provide ratings on a 4-point scale on symptoms over the past week. Final sum scores range from 0 to 30, with higher scores indicating greater levels of depression. Previous studies support both good internal consistency (α > 0.80) and validity of the EPDS [64]. The EPDS has been validated among Spanish-speaking mothers [65]. The EPDS is standardly administered at the prenatal and postpartum medical visits at Denver Health. We collect EPDS scores from participants’ electronic health records to address real-world feasibility and sustainability of depression monitoring.

#### 3.5.3. Implementation measures

Quantitative and qualitative measures of implementation process and outcomes guided by Proctor’s framework [57] and i-PARIHS model [58] are summarized in Table 1.

### 3.6. Analytic plan

#### 3.6.1. Analysis testing inferiority of virtual vs. in-person La Luz

**Fig. 2.**
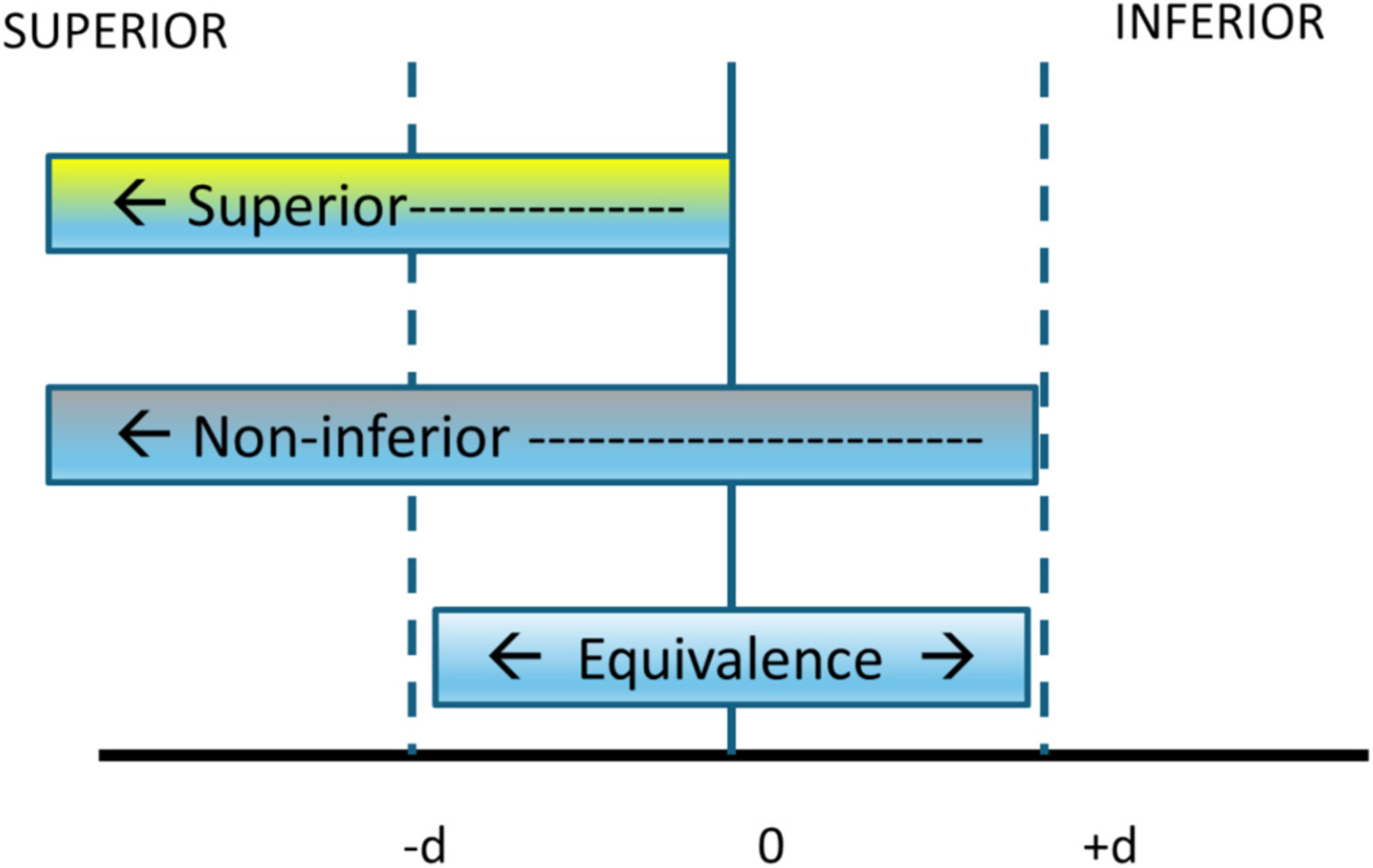
Superiority, non-inferiority, and equivalence trials. *Note*. This figure shows the relationship between superiority, non-inferiority, and equivalence trials. The negative and positive margins (−*d* and +*d)* indicate the confidence intervals set to establish the effect of the intervention.

Primary analyses will use intent-to-treat design to evaluate the effectiveness of the intervention among all enrolled participants [66]. To test whether virtual La Luz will yield a similar benefit (i.e., is non-inferior) as in-person La Luz for perinatal depression, multilevel models (timepoints nested within participants) including random intercepts and slopes will be used. Multilevel modeling accounts for nested data structure and is robust to missing data [67]. In line with non-inferiority testing, we will use a one-sided test and statistical significance set at *p* < .05 with margin of non-inferiority set to an effect size Cohen’s *d* of 0.25 for our estimate contrasts of total change in our multilevel model. Model-based total change estimate will provide us flexibility if the change over time is nonlinear (i.e., log-linear, parabolic, piecewise, etc.). A negative effect size would indicate more reduction for virtual compared to in-person, whereas, a positive effect size would indicate more reduction for in-person compared to virtual. Non-inferiority will be shown if the effect size for contrast of total change yields a one-sided 95 % upper bound smaller than *d* = +0.25. If the one-sided 95 % upper bound is bigger than *d* = +0.25, the confidence interval for the true effect size of the contrast of total change does not lie totally within the non-inferior region, and non-inferiority cannot be concluded (illustrated in Fig. 2 with ±*d* as the indicated margins). Given that intent-to-treat approaches can bias non-inferiority trials toward finding the interventions to be non-inferior, we will also conduct a sensitivity analysis including only participants who complete the intervention [68,69]. Secondary analyses will follow the same analytic procedures but with EPDS scores collected from electronic health records as the outcome.

### 3.7. Analysis of implementation process and outcomes

For qualitative analyses, we will use a rapid qualitative analysis [70], common in implementation science, to code the data, which is similar to thematic analysis in rigor but with greater efficiency [71]. A focused analysis of factors relevant for implementation will be conducted using a codebook informed by relevant research on perinatal depression, particularly among Black and Latine participants, Proctor’s implementation outcomes [57], and the i-PARIHS model [58]. After a first coding pass, a rapid-cycle evaluation approach will be used. After coding, we will generate reports from NVivo to compile all data segments by implementation outcome [70] and their correspondence to the iPAHRIS model components [71]. Analyses will also be conducted examining differential themes across participant characteristics (e.g., race, ethnicity) to ensure prevention programming does not inadvertently exacerbate existing health disparities.

With the quantitative data, we will examine implementation outcomes to identify areas of implementation weakness, separately by intervention modality. We will then explore themes in the qualitative data to gain additional insight into potential areas where implementation could be improved. We will organize identified barriers related to specific implementation outcomes and generate possible implementation strategies as recommendations for reducing barriers and enhancing implementation in other settings. We will organize these strategies and engage relevant stakeholders to develop a toolkit with specific recommendations for use in other settings [72].

## 4. Discussion

Perinatal depression is a highly prevalent complication of pregnancy [1], with higher rates among pregnant individuals who experience contextual stressors such as poverty and racism [11,12]. Perinatal depression not only negatively impacts the pregnant individuals and increases their risk for mortality [3,4], but it also has long-lasting adverse effects on the next generation [5–10]. Therefore, it is important to make effective and culturally sensitive prevention interventions for perinatal depression available for all pregnant individuals and address barriers to care. The current RCT tests the benefits of a novel implementation of ROSE (called La Luz in the current study)—delivering ROSE in a virtual format—which has only demonstrated efficacy when delivered in person. This study has a number of strengths. First, it represents the first effort to provide a novel direct and rigorous test of virtual versus in-person ROSE among socio-demographically high-risk pregnant individuals. Second, it delivers ROSE as universal prevention in a Federally Qualified Health Center through embedding ROSE in a hospital-based obstetric care setting. This universal prevention approach allows ROSE to have greater reach to pregnant individuals who can benefit from this intervention, particularly those who experience greater stigma and barriers to care. Relatedly, because ROSE has only been tested among those who are identified as at risk for post-partum depression based on prenatal screening [23,24,27,28], this study represents a novel test of the effectiveness of ROSE as universal prevention. Moreover, this study provides culturally sensitive care to pregnant individuals in their preferred language, both in-person and virtual intervention modalities, and necessary resources for participating in either modality (e.g., transportation, tablet). In particular, a substantial proportion of the recruited population identify as Hispanic or Latine [54], and many are Spanish-speaking and likely experience linguistic barriers [55]. Delivering ROSE in participants’ preferred language not only represents culturally responsive care but also provides the first empirical test of the effectiveness of ROSE delivered in Spanish. Lastly, the implementation science methods utilized in this research allow us to gain useful knowledge and insight from multiple stakeholders and offer great promise for building effective treatment tools for patients in a sustainable, scalable manner. The resultant stakeholder-informed toolkit of recommendations will support the program’s sustainability in Denver and scalability to other healthcare settings.

Alongside these strengths, the study includes design choices that impose some restrictions. For example, we intentionally elected to use a non-inferiority design, which uses two active treatment conditions to empirically test a delivery modality that can increase access to efficacious perinatal depression interventions (i.e., virtual delivery). This was because the literature demonstrates the benefit of ROSE and it would not be ethical to withhold the opportunity to participate. However, this design choice prevents us from testing whether either modality leads to superior clinical outcomes (e.g., lower depression) compared to a control group that does not receive ROSE in either modality. Additionally, although we had the same facilitators for in-person and virtual ROSE to keep facilitator-related factors consistent across conditions, it is possible that any preferences and beliefs facilitators may hold about either modality could inadvertently influence their delivery of ROSE and impact our findings. Lastly, because participants were randomly assigned to either virtual or in-person ROSE, they could not choose their preferred modality. Future research may investigate the effectiveness of ROSE when participants can freely receive their preferred modality, which more closely resembles real-world care.

Despite these restrictions, the current research critically contributes to the knowledge of perinatal depression prevention and addresses care access barriers of underserved populations. If effectiveness of virtual ROSE is demonstrated, the new evidence for a scalable low-cost preventive intervention can be used to reduce perinatal depression and offer accessible treatment options for people who face significant barriers to care. Future research should elucidate mechanisms of change, factors predicting differential impact of intervention, and sustainability and dissemination of virtual ROSE in various healthcare settings (e.g., non-hospital-based clinics).

## Figures and Tables

**Table 1:** Measurement of implementation progress and outcomes.

Proctor’s outcomes [57]	Definitions and measures
Acceptability	*Definition*: the perception that the intervention is agreeable, palatable, or satisfactory among stakeholders *Measures*: 4-item Acceptability of Intervention Measure [73] (e.g., “The program is appealing to me”)
Appropriateness	*Definition*: perceived fit, relevance, or compatibility of the intervention for a given practice setting, provider, or consumer or for addressing a particular issue *Measures*: 4-item Intervention Appropriateness Measure [73] (e.g., “The program seems like a good match for me.”)
Feasibility	*Definition*: the extent to which the intervention can be successfully carried out within a given agency or setting *Measures*: 4-item Feasibility of Intervention Measure [73] (e.g., The program seems doable to me.”) Recruitment metrics: time from study start to completion of recruitment, number of referrals to the study, and numbers of eligible and ineligible participants
Cost of implementation	*Definition*: cost impact of an implementation effort, which depends on depends on the costs of the intervention, the implementation strategy, and the location of service delivery. *Measures*: Structured interviews with personnel involved in administration of the intervention and assessment of resources to quantify cost of in-person and virtual ROSE Self-report data from patients on their use of all types of health services (inpatient, outpatient, pharmacy) EHR data from Denver Health on all types of service, service dates, beneficiary copayment, and primary and secondary ICD-10 and CPT4 codes for each episode of care over the 12-month period after completion of ROSE (after the postpartum booster) and for the 12-month period preceding pregnancy (and before participating in ROSE). Short-term increase in cost due to reductions of barriers to care evaluated via the Barriers to Access to Care Evaluation (BACE) [74], which is a 30-item comprehensive evaluation of barriers to obtaining mental health care and have good test-retest reliability and internal consistency.
i-PARIHS constructs [58]	Definition and measures
Innovation	*Definition*: assesses scientific research evidence against existing practice (e.g., the usability of the research evidence, its compatibility with existing policies and practices, its clarity) *Focus groups with the following example questions*: How well do you see the program meeting your needs? (for patients) What did you like about receiving the program in-person/virtually? (for patients) How well does the program fit into your existing services? (for administrators)
Recipients	*Definition*: takes a broad view of the individuals involved in the implementation process and includes patients/clients, providers, and clinic managers. *Focus groups with the following example questions*: How did the program address your cultural background? (for patients) How did the program fit into your daily life? (for patients) How did the program disrupt your usual work flow? (for providers)
Context	*Definition*: includes the immediate context (e.g., a clinical team, service, or clinic) and the outer context in which the clinical setting is embedded (e.g., the organizational infrastructures surrounding the clinic). *Focus groups with the following example questions*: Where would a program like this need to take place for you to feel interested and comfortable accessing it? (for patients) What things in your life got in the way of participating in the program in the way you would have liked? (for patients) What barriers do you see to maintain this program after the study ends? (for administrators)

*Note*. This table summarizes key implementation process and outcomes that will be assessed by the current study.

## Data Availability

No data was used for the research described in the article.

## References

[1] Underwood L, Waldie K, D’Souza S, Peterson ER, Morton S, A review of longitudinal studies on antenatal and postnatal depression, Arch. Womens Ment. Health 19 (5) (2016) 711–720, 10.1007/s00737-016-0629-1. 27085795

[2] Al-abri K, Edge D, Armitage CJ, Prevalence and correlates of perinatal depression, Soc. Psychiatry Psychiatr. Epidemiol 58 (11) (2023) 1581–1590, 10.1007/s00127-022-02386-9. 36646936 PMC9842219

[3] Wisner KL, Murphy C, Thomas MM, Prioritizing maternal mental health in addressing morbidity and mortality, JAMA Psychiatry 81 (5) (2024) 521–526, 10.1001/jamapsychiatry.2023.5648. 38381408

[4] Metz TD, Rovner P, Hoffman C, Allshouse AA, Beckwith KM, Binswanger IA, Maternal deaths from suicide and overdose in Colorado, 2004–2014, Obstet. Gynecol 128 (6) (2016) 1233–1240, 10.1097/AOG.0000000000001695. 27824771 PMC5121076

[5] Oyetunji A, Chandra P, Postpartum stress and infant outcome: a review of current literature, Psychiatry Res. 284 (2020) 112769, 10.1016/j.psychres.2020.112769. 31962260

[6] Accortt EE, Cheadle ACD, Dunkel Schetter C, Prenatal Depression and Adverse Birth Outcomes: An Updated Systematic Review vol. 19, Springer US, 2015, 10.1007/s10995-014-1637-2. PMC4447551 25452215

[7] Sandman CA, Buss C, Head K, Davis EP, Fetal exposure to maternal depressive symptoms is associated with cortical thickness in late childhood, Biol. Psychiatry 77 (4) (2015) 324–334, 10.1016/j.biopsych.2014.06.025. 25129235 PMC4289467

[8] Davis EP, Demers CH, Deer LB, , Impact of prenatal maternal depression on gestational length: post hoc analysis of a randomized clinical trial, eClinicalMedicine 72 (2024) 102601, 10.1016/j.eclinm.2024.102601. 38680516 PMC11053273

[9] Demers CH, Aran Ö, Glynn LM, Davis EP, Prenatal programming of neurodevelopment: structural and functional changes, in: Prenatal Stress and Child Development, 2021, pp. 193–242, 10.1007/978-3-030-60159-1_9.

[10] Urizar GG, Muñoz RF, Role of maternal depresson on child development: prospective analysis from pregnancy to early childhood, Child Psychiatry Hum. Dev 53 (3) (2022) 502–514, 10.1007/s10578-021-01138-1. 33646485 PMC10911822

[11] Hobfoll SE, Ritter C, Lavin J, Hulsizer MR, , Depression prevalence and incidence among inner-city pregnant and postpartum women, J. Consult. Clin. Psychol 63 (3) (1995) 445–453, 10.1037//0022-006x.63.3.445. 7608357

[12] Noroña-Zhou A, Aran Ö, Garcia SE, , Experiences of discrimination and depression trajectories over pregnancy, Women’s Heal Issues. 32 (2) (2022) 147–155, 10.1016/j.whi.2021.10.002. PMC9701536 34774402

[13] Liu CH, Tronick E, Rates and predictors of postpartum depression by race and ethnicity: results from the 2004 to 2007 New York city PRAMS survey (pregnancy risk assessment monitoring system), Matern. Child Health J 17 (9) (2013) 1599–1610, 10.1007/s10995-012-1171-z. 23095945

[14] Kozhimannil KB, Trinacty CM, Busch AB, Huskamp HA, Adams AS, Racial and ethnic disparities in postpartum depression care among low-income women, Psychiatr. Serv 62 (6) (2011) 619–625, 10.1007/s10995-012-1171-z. 21632730 PMC3733216

[15] Mukherjee S, Trepka MJ, Pierre-Victor D, Bahelah R, Avent T, Racial/ethnic disparities in antenatal depression in the United States: a systematic review, Matern. Child Health J 20 (9) (2016) 1780–1797, 10.1007/s10995-016-1989-x. 27016352

[16] Keyes KM, Martins SS, Hatzenbuehler ML, Blanco C, Bates LM, Hasin DS, Mental health service utilization for psychiatric disorders among Latinos living in the United States: the role of ethnic subgroup, ethnic identity, and language/social preferences, Soc. Psychiatry Psychiatr. Epidemiol 47 (3) (2012) 383–394, 10.1007/s00127-010-0323-y. 21290097 PMC3756540

[17] Vega WA, Karno M, Alegria M, , Research issues for improving treatment of U.S. Hispanics with persistent mental disorders, Psychiatr. Serv 58 (3) (2007) 385–394, 10.1176/ps.2007.58.3.385. 17325113

[18] ACOG Committee, ACOG Committee opinion no. 757: screening for perinatal depression, Obstet. Gynecol 132 (5) (2018) e208–e212. 30629567 10.1097/AOG.0000000000002927

[19] Davis EP, Hankin BL, Swales DA, Hoffman MC, An experimental test of the fetal programming hypothesis: can we reduce child ontogenetic vulnerability to psychopathology by decreasing maternal depression? Dev. Psychopathol 30 (3) (2018) 787–806, 10.1017/S0954579418000470. 30068416 PMC7040571

[20] Rhoades GK, Allen MOT, Peña R, Hyer J, Mazzoni SE, Relationship education for women during pregnancy: the impact of MotherWise on birth outcomes, Fam. Process 61 (3) (2022) 1134–1143, 10.1111/famp.12756. 35146754

[21] Hankin BL, Demers CH, Hennessey EMP, , Effect of brief interpersonal therapy on depression during pregnancy: a randomized clinical trial, JAMA Psychiatry 80 (6) (2023) 539–547, 10.1001/jamapsychiatry.2023.0702. 37074698 PMC10116385

[22] Le HN, Perry DF, Stuart EA, Randomized controlled trial of a preventive intervention for perinatal depression in high-risk latinas, J. Consult. Clin. Psychol 79 (2) (2011) 135–141, 10.1037/a0022492. 21319897

[23] Zlotnick C, Tzilos G, Miller I, Seifer R, Stout R, Randomized controlled trial to prevent postpartum depression in mothers on public assistance, J. Affect. Disord 189 (2016) 263–268, 10.1016/j.jad.2015.09.059. 26454186 PMC4641029

[24] Zlotnick C, Miller IW, Pearlstein T, Howard M, Sweeney P, A preventive intervention for pregnant women on public assistance at risk for postpartum depression, Am. J. Psychiatry 163 (8) (2006) 1443–1445, 10.1176/ajp.2006.163.8.1443. 16877662 PMC4387544

[25] Tandon SD, Leis JA, Mendelson T, Perry DF, Kemp K, Six-month outcomes from a randomized controlled trial to prevent perinatal depression in low-income home visiting clients, Matern. Child Health J 18 (4) (2014) 873–881, 10.1007/s10995-013-1313-y. 23793487 PMC3871944

[26] Ponting C, Mahrer NE, Zelcer H, Dunkel Schetter C, Chavira DA, Psychological interventions for depression and anxiety in pregnant Latina and black women in the United States: a systematic review, Clin. Psychol. Psychother 27 (2) (2020) 249–265, 10.1002/cpp.2424. 31960525 PMC7125032

[27] Zlotnick C, Capezza NM, Parker D, An interpersonally based intervention for low-income pregnant women with intimate partner violence: a pilot study, Arch. Womens Ment. Health 14 (1) (2011) 55–65, 10.1007/s00737-010-0195-x. 21153559 PMC3042850

[28] Zlotnick C, Johnson SL, Miller IW, Pearlstein T, Howard M, Postpartum depression in women receiving public assistance: pilot study of an interpersonal-therapy-oriented group intervention, Am. J. Psychiatry 158 (4) (2001) 638–640, 10.1176/appi.ajp.158.4.638. 11282702

[29] O’ConnorE, SengerCA, HenningerML, CoppolaE, GaynesBN, Interventions to prevent perinatal depression: evidence report and systematic review for the US preventive services task force, JAMA 321 (6) (2019) 588–601, 10.1001/jama.2018.20865.30747970

[30] Misra S, Jackson VW, Chong J, , Systematic review of cultural aspects of stigma and mental illness among racial and ethnic minority groups in the United States: implications for interventions, Am. J. Community Psychol 68 (3–4) (2021) 486–512, 10.1002/ajcp.12516. 33811676

[31] Alegría M, Chatterji P, Wells K, , Disparity in depression among ethnic populations, Psychiatr. Serv 59 (11) (2008) 1264–1272. 18971402 10.1176/appi.ps.59.11.1264 PMC2668139

[32] Miller ML, Dupree J, Monette MA, Lau EK, Peipert A, Health equity and perinatal mental health, Curr. Psychiatry Rep (2024), 10.1007/s11920-024-01521-4, 0123456789. 39008146

[33] Cook BL, Trinh NH, Li Z, Hou SSY, Progovac AM, Trends in racial-ethnic disparities in access to mental health care, 2004–2012, Psychiatr. Serv 68 (1) (2017) 9–16, 10.1176/appi.ps.201500453. 27476805 PMC5895177

[34] McGregor B, Li C, Baltrus P, , Racial and ethnic disparities in treatment and treatment type for depression in a national sample of medicaid recipients, Psychiatr. Serv 71 (7) (2020) 663–669, 10.1176/appi.ps.201900407. 32237981 PMC8842821

[35] Mendelson T, Rehkopf DH, Kubzansky LD, Depression among Latinos in the United States: a meta-analytic review, J. Consult. Clin. Psychol 76 (3) (2008) 355–366, 10.1037/0022-006X.76.3.355. 18540730

[36] Alegría M, Mulvaney-Day N, Woo M, Torres M, Gao S, Oddo V, Correlates of past-year mental health service use among Latinos: results from the National Latino and Asian American study, Am. J. Public Health 97 (1) (2007) 76–83, 10.2105/AJPH.2006.087197. 17138911 PMC1716237

[37] Alegría M, Canino G, Ríos R, , Inequalities in use of specialty mental health services among Latinos, African Americans, and non-Latino whites, Psychiatr. Serv 53 (12) (2002) 1547–1555, 10.1176/appi.ps.53.12.1547. 12461214

[38] Thomeer MB, Moody MD, Yahirun J, Racial and ethnic disparities in mental health and mental health care during the COVID-19 pandemic, J. Racial Ethn. Heal Disparities 10 (2) (2023) 961–976, 10.1007/s40615-022-01284-9. PMC8939391 35318615

[39] Webb R, Uddin N, Constantinou G, , Meta-review of the barriers and facilitators to women accessing perinatal mental healthcare, BMJ Open 13 (7) (2023), 10.1136/bmjopen-2022-066703. PMC10360426 37474171

[40] Ormel J, Cuijpers P, Jorm AF, Schoevers R, Prevention of depression will only succeed when it is structurally embedded and targets big determinants, World Psychiatry 18 (1) (2019) 111–112, 10.1002/wps.20580. 30600627 PMC6313244

[41] Byhoff E, Dinh DH, Lucas JA, Marino M, Heintzman J, Mental health care use by ethnicity and preferred language in a national cohort of community health center patients, Psychiatr. Serv 75 (4) (2024) 363–368, 10.1176/appi.ps.20220585. 37880967 PMC10984775

[42] Jones N, Marks R, Ramirez R, Ríos-Vargas M, 2020 Census Illuminates Racial and Ethnic Composition of the Country, United States Census Bureau, 2021. Published, https://www.census.gov/library/stories/2021/08/improved-race-ethnicity-measures-reveal-united-states-population-much-more-multiracial.html.

[43] United States Census Bureau. Explore Census Data. https://data.census.gov/table/ACSST1Y2023.S1601?q=LanguageSpokenatHome.

[44] March of Dimes, Fertility rates by race/ethnicity: United States, 2021–2023 Average, Published, https://www.marchofdimes.org/peristats/data?reg=99&top=2&stop=4&lev=1&slev=1&obj=1, 2024.

[45] Werner E, Le HN, Babineau V, Grubb M, Preventive interventions for perinatal mood and anxiety disorders: a review of selected programs, Semin. Perinatol 48 (6) (2024) 151944, 10.1016/j.semperi.2024.151944. 39048416

[46] Sultana S, Pagán JA, Use of telehealth to address depression and anxiety in low-income US populations: a narrative review, J. Prim Care Commun. Heal 14 (2023) 1–9, 10.1177/21501319231168036. PMC10134158 37096825

[47] Gamoran J, Xu Y, Buinewicz SAP, , An examination of depression severity and treatment adherence among racially and ethnically minoritized, low-income individuals during the COVID-19 transition to telehealth, Psychiatry Res. 342 (October) (2024) 116221, 10.1016/j.psychres.2024.116221. 39378538

[48] Phipps MG, Raker CA, Ware CF, Zlotnick C, Randomized controlled trial to prevent postpartum depression in adolescent mothers, Am. J. Obstet. Gynecol 208 (3) (2013) 192.e1–192.e6, 10.1016/j.ajog.2012.12.036. PMC4386618 23313720

[49] Crockett K, Zlotnick C, Davis M, Payne N, Washington R, A depression preventive intervention for rural low-income African-American pregnant women at risk for postpartum depression, Arch. Womens Ment. Health 11 (5–6) (2008) 319–325, 10.1007/s00737-008-0036-3. 18982408

[50] Hanach N, de Vries N, Radwan H, Bissani N, The effectiveness of telemedicine interventions, delivered exclusively during the postnatal period, on postpartum depression in mothers without history or existing mental disorders: a systematic review and meta-analysis, Midwifery 94 (2021) 102906, 10.1016/j.midw.2020.102906. 33360589

[51] Zhao L, Chen J, Lan L, , Effectiveness of telehealth interventions for women with postpartum depression: systematic review and meta-analysis, JMIR Mhealth Uhealth 9 (10) (2021) e32544, 10.2196/32544. 34617909 PMC8532017

[52] Denver Health. Providing High-Quality Cost-Effective Care for all since 1860. https://www.denverhealth.org/-/media/images/content-images/about/denver-health-providing-high-quality-cost-effective-care.pdf?la=en&hash=5140FBBCB1E6EF1250902C16C2F80D37987C27EB.

[53] Denver Health, Maternity and Pregnancy, Published, https://www.denverhealth.org/services/obstetrics-and-gynecology/maternity-pregnancy, 2025.

[54] Denver Health, Denver Health: Report to the City. https://www.denverhealth.org/-/media/2023-denverhealth-reporttothecity, 2023.

[55] Hernandez EL, Denver’s Spanish-Speaking Doctors Are Fighting COVID-19 with their Language Skills. Their Care Goes Beyond Words, Published, https://serviciosdelaraza.org/denvers-spanish-speaking-doctors-are-fighting-covid-19-with-their-language-skills-their-care-goes-beyond-words/, 2021.

[56] Women & Infants Hospital. What Is the ROSE Program? An Evidence-Based Intervention to Prevent Postpartum Depression. https://www.womenandinfants.org/rose-program-postpartum-depression.

[57] Proctor E, Silmere H, Raghavan R, , Outcomes for implementation research: conceptual distinctions, measurement challenges, and research agenda, Adm. Policy Ment. Heal. Ment. Heal. Serv. Res 38 (2) (2011) 65–76, 10.1007/s10488-010-0319-7. PMC3068522 20957426

[58] Harvey G, Kitson A, PARIHS revisited: from heuristic to integrated framework for the successful implementation of knowledge into practice, Implement. Sci 11 (1) (2016), 10.1186/s13012-016-0398-2. PMC4807546 27013464

[59] Derogatis LR, Lipman RS, Rickels K, Uhlenhuth EH, Covi L, The Hopkins symptom checklist (HSCL): a self-report symptom inventory, Behav. Sci 19 (1) (1974) 1–15, 10.1002/bs. 3830190102. 4808738

[60] Björgvinsson T, Kertz SJ, Bigda-Peyton JS, McCoy KL, Aderka IM, Psychometric properties of the CES-D-10 in a psychiatric sample, Assessment 20 (4) (2013) 429–436, 10.1177/1073191113481998. 23513010

[61] Radloff LS, The CES-D scale: a self-report depression scale for research in the general population, Appl. Psychol. Measur 1 (3) (1977) 385–401.

[62] Grote NK, Katon WJ, Russo JE, , A randomized trial of collaborative care for perinatal depression in socioeconomically disadvantaged women: the impact of comorbid posttraumatic stress disorder, J. Clin. Psychiatry 77 (11) (2016) 1527–1537, 10.4088/JCP.15m10477. 28076671

[63] Bergink V, Kooistra L, Lambregtse-van den Berg MP, , Validation of the Edinburgh depression scale during pregnancy, J. Psychosom. Res 70 (4) (2011) 385–389, 10.1016/j.jpsychores.2010.07.008. 21414460

[64] Cox JL, Holden JM, Sagovsky R, Detection of postnatal depression: development of the 10-item Edinburgh postnatal depression scale, Br. J. Psychiatry 150 (1987) 782–786, 10.1192/bjp.150.6.782. 3651732

[65] Garcia-Esteve L, Ascaso C, Ojuel J, Navarro P, Validation of the Edinburgh postnatal depression scale (EPDS) in Spanish mothers, J. Affect. Disord 75 (1) (2003) 71–76, 10.1016/S0165-0327(02)00020-4. 12781353

[66] Lachin JM, Statistical considerations in the intent-to-treat principle, Control. Clin. Trials 21 (2000) 167–189, 10.1016/S0197-2456(00)00046-5. 10822117

[67] Raudenbush SW, Bryk AS, Hierarchical Linear Models: Applications and Data Analysis Methods, 2nd ed, Sage, 2002.

[68] Walker E, Nowacki AS, Understanding equivalence and noninferiority testing, J. Gen. Intern. Med 26 (2) (2011) 192–196, 10.1007/s11606-010-1513-8. 20857339 PMC3019319

[69] Head SJ, Kaul S, Bogers AJJC, Kappetein AP, Non-inferiority study design: lessons to be learned from cardiovascular trials, Eur. Heart J 33 (11) (2012) 1318–1324, 10.1093/eurheartj/ehs099. 22564354

[70] Hamilton AB, Finley EP, Qualitative methods in implementation research: an introduction, Psychiatry Res. 280 (2019) 112516, 10.1016/j.psychres.2019.112516. 31437661 PMC7023962

[71] Taylor B, Henshall C, Kenyon S, Litchfield I, Greenfield S, Can rapid approaches to qualitative analysis deliver timely, valid findings to clinical leaders? A mixed methods study comparing rapid and thematic analysis, BMJ Open 8 (10) (2018) 5–7, 10.1136/bmjopen-2017-019993. PMC6194404 30297341

[72] Lewis CC, Scott K, Marriott BR, A methodology for generating a tailored implementation blueprint: an exemplar from a youth residential setting, Implement. Sci 13 (1) (2018) 1–13, 10.1186/s13012-018-0761-6. 29769096 PMC5956960

[73] Weiner BJ, Lewis CC, Stanick C, , Psychometric assessment of three newly developed implementation outcome measures, Implement. Sci 12 (1) (2017) 1–12, 10.1186/s13012-017-0635-3. 28851459 PMC5576104

[74] Clement S, Brohan E, Jeffery D, Henderson C, Hatch SL, Thornicroft G, Development and psychometric properties the barriers to access to care evaluation scale (BACE) related to people with mental ill health, BMC Psychiatry 12 (1) (2012), 10.1186/1471-244X-12-36. PMC3379935 22546012

